# Supplementary material for: Translation and cultural adaptation of drug use stigma and HIV stigma measures among people who use drugs in Tanzania
Source: PLoS One. 2023 Oct 19;18(10):e0292642. doi: 10.1371/journal.pone.0292642 (PMC10586607; doi:10.1371/journal.pone.0292642)
Supplement: S1 File — (DOCX) [file pone.0292642.s001.docx]

**S1 File. Forward and back translated HIV and drug use stigma measures**

1. **Abridged Berger Scale – Validated in India – Original English (OE)**

**Abridged Berger Scale –Imethibitishwa India – Reconciled Forward translation (RFT)**

**Abridged Berger Scale –Certified by India – Back translated version 1 (BTV1)**

**Abridged Berger Scale –Certified in India – Back translated version 2 (BTV2)**

**Response options:**

| OE | Strongly disagree | Disagree | Agree | Strongly agree |
| --- | --- | --- | --- | --- |
| RFT | Sikubali Kabisa | Sikubali | Nakubali | Nakubali kabisa |
| BTVI | Strongly disagree | Disagree | Agree | Strongly agree |
| BTV2 | Strongly Disagree | Disagree | Agree | Strongly Disagree |

**Items**

| **Sn** | OE | Item |
| --- | --- | --- |
|  | RFT | Kipengele |
|  | BTVI | Category |
|  | BTV2 | Aspect |
|  | OE | **Personalized stigma** |
|  | RFT | **Unyanyapaa binafsi** |
|  | BTVI | **Self-Stigmatization** |
|  | BTV2 | **Personal Stigma** |
| 1 | OE | Some people close to me are afraid others will reject them if it becomes known that I have HIV |
|  | RFT | Baadhi ya watu wangu wa karibu wanaogopa kukataliwa na watu wengine endapo itajulikana nina VVU |
|  | BTVI | Certain people who are close to me are fearful of not being accepted by other people if it is discovered that I have HIV |
|  | BTV2 | My close associates are worried that they may be rejected by other people if it is discovered that I am HIV-positive |
| 2 | OE | People have physically backed away from me when they learn I have HIV |
|  | RFT | Watu wamejitenda nami baada ya kufahamu kuwa nina VVU |
|  | BTVI | People have distanced themselves from me after finding out I have HIV |
|  | BTV2 | People have avoided me after discovering that I am HIV-positive |
| 3 | OE | People who know I have HIV tend to ignore my good points |
|  | RFT | Watu wanaojua nina VVU wamekuwa wakipuuza maoni yangu mazuri |
|  | BTVI | All my good ideas have been undermined by people who know I have HIV |
|  | BTV2 | People who know that I am HIV-positive tend to ignore my positive views |
| 4 | OE | Some people avoid touching me once they know I have HIV |
|  | RFT | Baadhi ya watu hukwepa kunigusa pale wanapojua nina VVU |
|  | BTVI | Certain people avoid touching me once they know I have HIV |
|  | BTV2 | Some people avoid contact with me when they know that I am HIV-positive |
| 5 | OE | I have stopped socializing with some people because of their reactions to my having HIV |
|  | RFT | Nimeacha kujumuika na baadhi ya watu kwa sababu ya namna wanavyochukulia hali yangu ya VVU |
|  | BTVI | I have stopped socializing with certain people because of how they react to my HIV status |
|  | BTV2 | I have stopped socializing with some people because of the way they perceive my HIV status |
| 6 | OE | People I care about stopped calling after learning I have HIV |
|  | RFT | Watu ninaowajali wameacha kunipigia simu baada ya kugundua nina VVU |
|  | BTVI | People I care about have stopped calling me after discovering I have HIV |
|  | BTV2 | The people I care have stopped calling me after discovering that I am HIV-positive |
| 7 | OE | People seem afraid of me once they learn I have HIV |
|  | RFT | Watu huonesha kuniogopa mara baada ya kugundua nina VVU |
|  | BTVI | People appear scared of me once they discover I have HIV |
|  | BTV2 | People tend to be afraid of me once they discover that I am HIV-positive |
| 8 | OE | I have been hurt by how people reacted to learning I have HIV |
|  | RFT | Nimekuwa nikiumizwa na namna watu wanavyochukulia taarifa kuwa nina VVU |
|  | BTVI | I have been hurt by peoples’ reaction to the news that I have HIV |
|  | BTV2 | I have been troubled by the way people perceive the news that I am HIV-positive |
| 9 | OE | People don’t want me around their children once they know I have HIV |
|  | RFT | Watu hawataki niwe karibu na watoto wao mara wanapojua nina VVU |
|  | BTVI | People do not want me to be near their children once they find out I have HIV |
|  | BTV2 | People do not like me to be close to their children once they discover that I am HIV-positive |
| 10 | OE | I have lost friends by telling them I have HIV |
|  | RFT | Nimepoteza marafiki baada ya kuwaambia kuwa nina VVU |
|  | BTVI | I have lost friends after telling them I have HIV |
|  | BTV2 | I have lost friends after telling them that I am HIV-positive |
| 11 | OE | Some people who know I have HIV have grown more distant |
|  | RFT | Baadhi ya watu wanaofahamu kuwa nina VVU wamejitenga na mimi |
|  | BTVI | I have also been distanced by certain people who know I have HIV |
|  | BTV2 | Some of the people who know that I am HIV-positive have excommunicated me |
|  | OE | **Negative self-image** |
|  | RFT | **Taswira/Mwonekano binafsi hasi** |
|  | BTVI | **Appearance/Negative self-image** |
|  | BTV2 | **Image/personal negative perceptions** |
| 12 | OE | Having HIV makes me feel unclean |
|  | RFT | Kuwa na VVU hunifanya nijihisi sio msafi/mdhambi |
|  | BTVI | Having HIV makes me feel dirty/sinful |
|  | BTV2 | My HIV status makes me feel that I am unclean/sinful |
| 13 | OE | Having HIV makes me feel that I’m a bad person |
|  | RFT | Kuwa na VVU hunifanya nijihisi kuwa mie ni mtu mbaya |
|  | BTVI | Having HIV makes me feel like a bad person |
|  | BTV2 | My HIV status makes me feel that I am evil |
| 14 | OE | People’s attitudes about HIV make me feel worse about myself. |
|  | RFT | Mitazamo ya watu kuhusu VVU hunifanya nijisikie vibaya zaidi |
|  | BTVI | Peoples’ opinions about HIV makes me feel worse |
|  | BTV2 | People’s perceptions of HIV make me feel worse |
| 15 | OE | I feel I am not as good a person as others because I have HIV |
|  | RFT | Nahisi mimi sio mtu mzuri kama wengine kwasabu nina VVU |
|  | BTVI | I feel I am not a good person like other people because I have HIV |
|  | BTV2 | I feel that I am not as good person as others because I am HIV-positive |
| 16 | OE | Some people act as though it’s my fault that I have HIV |
|  | RFT | Baadhi ya watu wanachukulia kama ni kosa langu kuwa na VVU |
|  | BTVI | Certain people believe that it is my fault that I have HIV |
|  | BTV2 | Some people perceive that I am to blame for my HIV status |
| 17 | OE | Since learning I have HIV, I feel set apart and isolated from the rest of world |
|  | RFT | Tangu nimefahamu kuwa nina VVU, najihisi kutengwa na kuwa mbali na ulimwengu |
|  | BTVI | Since finding out I have HIV, I feel isolated from the world |
|  | BTV2 | Since I discovered that I am HIV-positive, I feel to be excommunicated and kept away from the world |
|  | OE | **Public attitudes** |
|  | RFT | **Mitazamo ya jamii** |
|  | BTVI | **Social Attitudes** |
|  | BTV2 | **Social perceptions** |
| 18 | OE | Most people believe that a person who has HIV is dirty |
|  | RFT | Watu wengi wanaamini kuwa mtu mwenye VVU ni mchafu/mdhambi |
|  | BTVI | Many people believe that a person with HIV is dirty/sinful |
|  | BTV2 | Most people believe that an HIV-positive person is filthy/sinful |
| 19 | OE | Most people with HIV are rejected when others find out |
|  | RFT | Watu wengi wenye VVU hukataliwa pindi watu wanapogundua hali zao |
|  | BTVI | Most people with HIV are rejected when people discover their status |
|  | BTV2 | Most people with HIV are rejected when people discover their HIV status |
| 20 | OE | People with HIV lose their jobs when their employers find out |
|  | RFT | Watu wenye VVU hupoteza kazi zao pindi waajiri wao wanapogundua hali zao |
|  | BTVI | People with HIV lose their jobs when their employers find out their status |
|  | BTV2 | People with HIV lose their jobs when employers discover their HIV status |
| 21 | OE | Most people think that a person with HIV is disgusting |
|  | RFT | Watu wengi hudhani kuwa mtu mwenye VVU anakera |
|  | BTVI | Many people think that a person with HIV is irritating |
|  | BTV2 | Most people think that a person with HIV is a bore |
|  | OE | **Disclosure concerns** |
|  | RFT | **Masuala ya kuweka wazi** |
|  | BTVI | **Issue to be Clarified** |
|  | BTV2 | **Issues pertaining to transparency** |
| 22 | OE | I never feel the need to hide the fact that I have HIV |
|  | RFT | Sioni haja ya kuficha ukweli kuwa nina VVU |
|  | BTVI | I do not see the need to hide the fact that I am HIV positive |
|  | BTV2 | I don’t see why I should hide the truth that I am HIV-positive |
| 23 | OE | I worry that people who know I have HIV will tell others |
|  | RFT | Ninahofia kuwa watu wanaojua nina VVU watawambia wengine |
|  | BTVI | I am afraid people who know I have HIV will tell others |
|  | BTV2 | I am worried that people who know that I am HIV-positive may tell others |
| 24 | OE | I regret having told some people that I have HIV |
|  | RFT | Ninajuta kuwaambia baadhi ya watu kuwa nina VVU |
|  | BTVI | I regret telling certain people that I have HIV |
|  | BTV2 | I wish that I did not tell some people that I am HIV-positive |
| 25 | OE | I worry that people may judge me when they learn I have HIV |
|  | RFT | Ninahofia watu wanaweza kunihukumu watakapogundua nina VVU |
|  | BTVI | I am afraid that people may judge me once they find out that I have HIV |
|  | BTV2 | I am worried that people may blame me if they discover that I am HIV-positive |

1. **Internalized AIDS-Related Stigma Scale (IA-RSS) - OE**

**Kipimo cha unyanyapaa binafsi kuhusiana na UKIMWI – RFT**

**Measure of personal stigma related to AIDS – BTV1**

**Scale for personal stigma in relation to HIV – BTV2**

**Response options**

| OE | **Agree** | **Disagree** |
| --- | --- | --- |
| RFT | **Nakubali** | **Sikubali** |
| BTVI | **Agree** | **Disagree** |
| BTV2 | **Agree** | **Disagree** |

**Items**

| **Sn** | OE | **Item** |
| --- | --- | --- |
|  | RFT | **Kipengele** |
|  | BTVI | **Category** |
|  | BTV2 | **Aspect** |
| 1 | OE | It is difficult to tell people about my HIV infection |
|  | RFT | Ni vigumu kuwaambia watu juu ya hali yangu ya maambukizi ya VVU |
|  | BTVI | It is difficult to tell people about my HIV status |
|  | BTV2 | It is difficult to tell people about my status regarding HIV infections |
| 2 | OE | being HIV positive makes me feel dirty |
|  | RFT | Kuwa na VVU kunanifanya nijihisi mchafu/mdhambi |
|  | BTVI | Having HIV makes me feel dirty/sinful |
|  | BTV2 | My HIV status makes me feel that I am filthy/ sinful |
| 3 | OE | I feel guilty that I am HIV positive |
|  | RFT | Najihisi mwenye hatia kwa kuwa nina VVU |
|  | BTVI | I feel guilty for being HIV positive |
|  | BTV2 | I feel guilty because I am HIV-positive |
| 4 | OE | I am ashamed that I am HIV positive |
|  | RFT | Ninajisikia aibu kwa kuwa nina VVU |
|  | BTVI | I feel ashamed because I am HIV positive |
|  | BTV2 | I feel shy because I am HIV-positive |
| 5 | OE | I sometimes feel worthless because I am HIV positive |
|  | RFT | Wakati mwingine najihisi sina thamani kwasababu nina VVU |
|  | BTVI | Sometimes I feel worthless because I am HIV positive |
|  | BTV2 | Sometimes I feel devalued because I am HIV-positive |
| 6 | OE | I hide my HIV status from others |
|  | RFT | Ninaficha hali yangu ya maambukizi ya VVU kwa watu wengine |
|  | BTVI | I hide my HIV status from some people |
|  | BTV2 | I hide my HIV status from other people |

1. **HIV Stigma Mechanism Measure - OE**

**Kipimo cha unyanyapaa dhidi ya VVU – RFT**

**Measure of stigma against HIV – BTV1**

**Scale for HIV stigma – BTV2**

**Response options**

| OE | **How do you feel about being HIV-positive?** | | | | |
| --- | --- | --- | --- | --- | --- |
| RFT | Unajisikiaje kuathirika na VVU? | | | | |
| BTVI | How do you feel about being infected with HIV? | | | | |
| BTV2 | How do you feel about your HIV status? | | | | |
| OE | **Strongly disagree** | **Disagree** | **Neither disagree nor agree** | **Agree** | **Strongly agree** |
| RFT | **Sikubali kabisa** | **Sikubali** | **Sikubali wala**  **sikatai** | **Nakubali** | **Nakubali kabisa** |
| BTVI | **Strongly Disagree** | **Disagree** | **Neutral** | **Agree** | **Strongly Agree** |
| BTV2 | **Strongly Disagree** | **Disagree** | **Neither disagree nor agree** | **Agree** | **Strongly agree** |

**Items**

| **Sn** | OE | **Statement** | | | | | |
| --- | --- | --- | --- | --- | --- | --- | --- |
|  | RFT | **Maelezo** | | | | | |
|  | BTVI | **Details** | | | | | |
|  | BTV2 | **Statements** | | | | | |
| 1 | OE | Having HIV makes me feel like I’m a bad person | | | | | |
|  | RFT | Kuwa na VVU kunanifanya nijihisi kuwa ni mtu mbaya | | | | | |
|  | BTVI | Having HIV makes me feel like a bad person | | | | | |
|  | BTV2 | Being HIV-positive makes me feel that I a bad person | | | | | |
| 2 | OE | I feel I’m not as good as others because I have HIV | | | | | |
|  | RFT | Najihisi sio mtu mzuri kama wengine kwasababu nina VVU | | | | | |
|  | BTVI | I feel I am not a good person like others because I have HIV | | | | | |
|  | BTV2 | I feel that I am not as good person as others because I am HIV-positive | | | | | |
| 3 | OE | I feel ashamed of having HIV | | | | | |
|  | RFT | Ninajisikia aibu kuwa na VVU | | | | | |
|  | BTVI | I feel ashamed of having HIV | | | | | |
|  | BTV2 | I feel shy because I am HIV-positive | | | | | |
| 4 | OE | I think less of myself because I have HIV | | | | | |
|  | RFT | Najishusha thamani kwasabu nina VVU | | | | | |
|  | BTVI | I lower my value because I have HIV | | | | | |
|  | BTV2 | I devalue myself because I am HIV-positive | | | | | |
| 5 | OE | Having HIV makes me feel unclean | | | | | |
|  | RFT | Kuwa na VVU hunifanya nijihisi sio msafi/mdhambi | | | | | |
|  | BTVI | Having HIV makes me feel dirty/sinful | | | | | |
|  | BTV2 | Being HIV-positive makes me feel that I am unclean/sinful | | | | | |
| 6 | OE | Having HIV is disgusting to me | | | | | |
|  | RFT | Kuwa na VVU ni kero kwangu | | | | | |
|  | BTVI | Having HIV is irritating to me | | | | | |
|  | BTV2 | Being HIV-positive is boring to me | | | | | |
|  | OE | **How likely is it that people will treat you in the following ways in the future because of your HIV status?** | | | | | |
|  | RFT | **Ni kwa namna gani watu watakuchukulia baadae katika njia zifuatazo kwasababu ya hali yako ya maambukizi ya VVU?** | | | | | |
|  | BTVI | **How will people treat you later in the following ways because of your HIV status?** | | | | | |
|  | BTV2 | **How shall people perceive you this way in the future because of your HIV status?** | | | | | |
|  | **Response options** | | | | | | |
|  | OE | **Very unlikely** | **Unlikely** | **Neither unlikely nor likely** | **Likely** | | **Very likely** |
|  | RFT | **Haiwezekani kabisa** | **Haiwezekani** | **Sina hakika** | **Inawezekana** | | **Inawezekana sana** |
|  | BTVI | **Not possible at all** | **Not possible** | **Not sure** | **Possible** | | **Very Possible** |
|  | BTV2 | **Quite impossible** | **Impossible** | **Not sure** | **Possible** | | **Quite possible** |
|  | OE | **Statements** | | | | | |
|  | RFT | **Maelezo** | | | | | |
|  | BTVI | **Details** | | | | | |
|  | BTV2 | **Statements** | | | | | |
| 1 | OE | Family members will avoid me | | | | | |
|  | RFT | Wanafamilia watanikwepa | | | | | |
|  | BTVI | Family members will avoid me | | | | | |
|  | BTV2 | Family members shall avoid me | | | | | |
| 2 | OE | Family members will look down on me | | | | | |
|  | RFT | Wanafamilia watanidharau | | | | | |
|  | BTVI | Family members will despise me | | | | | |
|  | BTV2 | Family members shall despise me | | | | | |
| 3 | OE | Family members will treat me differently | | | | | |
|  | RFT | Wanafamilia watanichukulia tofauti | | | | | |
|  | BTVI | Family members will treat me differently | | | | | |
|  | BTV2 | Family members shall perceive me differently | | | | | |
| 4 | OE | Community/social workers won’t take my needs seriously | | | | | |
|  | RFT | Jamii/ afisa ustawi wa jamii hawatachukulia mahitaji yangu kwa umakini | | | | | |
|  | BTVI | The community/social welfare officer will not take my needs seriously | | | | | |
|  | BTV2 | Society/social welfare officer shall not take my needs seriously | | | | | |
| 5 | OE | Community/social workers will discriminate against me | | | | | |
|  | RFT | Jamii/ afisa ustawi wa jamii watanibagua | | | | | |
|  | BTVI | The community/social welfare officer will discriminate against me | | | | | |
|  | BTV2 | Society/social welfare officer shall discriminate me against | | | | | |
| 6 | OE | Community/social workers will deny me services | | | | | |
|  | RFT | Jamii/ afisa ustawi wa jamii watakataa kunihudumia | | | | | |
|  | BTVI | The community/social welfare officer will refuse to treat me | | | | | |
|  | BTV2 | Society/ social welfare officer shall deny me services | | | | | |
| 7 | OE | Healthcare workers will not listen to my concerns | | | | | |
|  | RFT | Watoa huduma za afya hawatasikiliza haja zangu | | | | | |
|  | BTVI | Healthcare providers will not listen to my needs | | | | | |
|  | BTV2 | Health care providers shall not attend to my concerns | | | | | |
| 8 | OE | Healthcare workers will avoid touching me | | | | | |
|  | RFT | Watoa huduma za afya wataepuka kunigusa | | | | | |
|  | BTVI | Healthcare providers will avoid touching me | | | | | |
|  | BTV2 | Health care providers shall avoid contact with me | | | | | |
| 9 | OE | Healthcare workers will treat me with less respect | | | | | |
|  | RFT | Wahudumu wa afya watanihudumia bila heshima | | | | | |
|  | BTVI | Health workers will treat me with disrespect | | | | | |
|  | BTV2 | Health personnel shall serve me with contempt | | | | | |
|  | OE | **How often have people treated you this way in the past because of your HIV status?** | | | | | |
|  | RFT | **Ni mara ngapi watu wamekutendea hivi kipindi cha nyuma baada ya kujua hali yako ya maambukizi ya VVU?** | | | | | |
|  | BTVI | **How often have people treated you this way in the past because of your HIV status?** | | | | | |
|  | BTV2 | **How often have people treated you this way in the past because of your HIV status?** | | | | | |
|  | OE | **Statement** | | | | | |
|  | RFT | **Maelezo** | | | | | |
|  | BTVI | **Details** | | | | | |
|  | BTV2 | **Statements** | | | | | |
|  | OE | **Never** | **Not often** | **Somewhat often** | | **Often** | **Very often** |
|  | RFT | **Haijawahi kutokea** | **Sio mara nyingi** | **Mara kadhaa** | | **Mara nyingi** | **Mara nyingi sana** |
|  | BTVI | **Did not Happen** | **Not often** | **Sometimes** | | **Often** | **Frequently** |
|  | BTV2 | **Never** | **Not often** | **Several times** | | **Often** | **Very often** |
| 1 | OE | Family members have avoided me | | | | | |
|  | RFT | Wanafamilia wamenikwepa | | | | | |
|  | BTVI | Family members avoid me | | | | | |
|  | BTV2 | Family members have avoided me | | | | | |
| 2 | OE | Family members have looked down on me | | | | | |
|  | RFT | Wanafamilia wamenidharau | | | | | |
|  | BTVI | Family members despise me | | | | | |
|  | BTV2 | Family members have despised me | | | | | |
| 3 | OE | Family members have treated me differently | | | | | |
|  | RFT | Wanafamilia wamenichukulia tofauti | | | | | |
|  | BTVI | Family members treat me differently | | | | | |
|  | BTV2 | Family members have perceived me differently | | | | | |
| 4 | OE | Community /social workers have not taken my needs seriously | | | | | |
|  | RFT | Jamii/ afisa ustawi wa jamii hawajachukulia mahitaji yangu kwa umakini | | | | | |
|  | BTVI | The community/social welfare officer did not take my needs seriously | | | | | |
|  | BTV2 | Society/social welfare officer have not taken my needs seriously | | | | | |
| 5 | OE | Community /social workers have discriminated against me | | | | | |
|  | RFT | Jamii/ afisa ustawi wa jamii wamenibagua | | | | | |
|  | BTVI | The community/social welfare officer discriminated against me | | | | | |
|  | BTV2 | Society/ social welfare officer have discriminated me against | | | | | |
| 6 | OE | Community/social workers have denied me services | | | | | |
|  | RFT | Jamii/ afisa ustawi wa jamii wamekataa kunihudumia | | | | | |
|  | BTVI | The community/social welfare officer refused to treat me | | | | | |
|  | BTV2 | Society/social welfare officer have denied me services | | | | | |
| 7 | OE | Healthcare workers have not listened to my concersn | | | | | |
|  | RFT | Watoa huduma za afya hawajasikiliza haja zangu | | | | | |
|  | BTVI | Healthcare providers did not listen to my needs | | | | | |
|  | BTV2 | Health care providers have never attended to my concerns | | | | | |
| 8 | OE | Healthcare workers have avoided touching me | | | | | |
|  | RFT | Watoa huduma za afya wameepuka kunigusa | | | | | |
|  | BTVI | Healthcare providers avoided touching me | | | | | |
|  | BTV2 | Health care providers have avoided contact with me | | | | | |
| 9 | OE | Healthcare workers have treated me with less respect | | | | | |
|  | RFT | Watoa huduma za afya wameepuka kunigusa | | | | | |
|  | BTVI | Health workers will treat me with disrespect | | | | | |
|  | BTV2 | Health care providers have attended to me with contempt | | | | | |

1. **Internalized Drug use Stigma Scale - OE**

**Kiwango cha unyanyapaa binafsi katika matumizi ya dawa za kulevya – RFT**

**The level of personal stigma in drug use – BTV1**

**Degree of personal stigma for using drugs – BTV2**

**Response options**

| OE | **Strongly agree** | **Agree** | **Neither agree nor disagree** | **Disagree** | **Strongly disagree** |
| --- | --- | --- | --- | --- | --- |
| RFT | **Nakubali kabisa** | **nakubali** | **Sikubali wala sikatai** | **Sikubali** | **Sikubali kabisa** |
| BTVI | **Strongly agree** | **Agree** | **Neutral** | **Disagree** | **Strongly Disagree** |
| BTV2 | **Strongly agree** | **Agree** | **Neither Disagree nor agree** | **Disagree** | **Strongly disagree** |

**Items**

| **Sn** | OE | **Item** |
| --- | --- | --- |
|  | RFT | **Kipengele** |
|  | BTVI | **Category** |
|  | BTV2 | **Aspect** |
| 1 | OE | It is difficult to tell people about being a person who uses drugs |
|  | RFT | Ni vigumu kuwaambia watu kuwa unatumia dawa za kulevya |
|  | BTVI | It is hard to tell people that you are using drugs |
|  | BTV2 | It is difficult to tell people that you use drugs |
| 2 | OE | Using drugs makes me feel dirty |
|  | RFT | Kutumia dawa za kulevya kunanifanya nijihisi mchafu/mdhambi |
|  | BTVI | Using drugs makes me feel dirty/sinful |
|  | BTV2 | Use of drugs makes me feel filthy/sinful |
| 3 | OE | I feel guilty that I use drugs |
|  | RFT | Najihisi mwenye hatia kwa kuwa natumia dawa za kulevya |
|  | BTVI | I feel guilty for using drugs |
|  | BTV2 | I feel guilty because I use drugs |
| 4 | OE | I am ashamed that I use drugs |
|  | RFT | Najisikia aibu kwa kuwa natumia dawa za kulevya |
|  | BTVI | I feel shame for using drugs |
|  | BTV2 | I feel shy because I use drugs |
| 5 | OE | I sometimes feel worthless because I use drugs |
|  | RFT | Wakati mwingine najihisi sina thamani kwasababu natumia dawa za kulevya |
|  | BTVI | Sometimes I feel worthless because I use drugs |
|  | BTV2 | Sometimes I feel devalued because I use drugs |
| 6 | OE | I hide my status of using drugs from others |
|  | RFT | Naficha hali yangu ya matumizi ya dawa za kulevya kwa watu wengine |
|  | BTVI | I hide m use of drugs from some people |
|  | BTV2 | I hide my status regarding use of drugs from other people |

1. **Substance use stigma mechanism model - OE**

**Mfano wa utaratibu wa unyanyapaa kwa matumizi ya dawa za kulevya – RFT**

**An example of systematic stigma for drug use – BTV1**

**Examples of stigma practices for use of drugs – BTV2**

**Response options**

| OE | **Strongly disagree** | **Disagree** | **Neither disagree nor agree** | **Agree** | **Strongly agree** |
| --- | --- | --- | --- | --- | --- |
| RFT | **Sikubali kabisa** | **Sikubali** | **Sikubali wala sikatai** | **Nakubali** | **Nakubali kabisa** |
| BTVI | **Strongly Disagree** | **Disagree** | **Neutral** | **Agree** | **Strongly Agree** |
| BTV2 | **Strongly disagree** | **Disagree** | **Neither disagree nor agree** | **Agree** | **Strongly agree** |

**Items**

| **Sn** | OE | **Item** |
| --- | --- | --- |
|  | RFT | **Kipengele** |
|  | BTVI | **Category** |
|  | BTV2 | **Aspect** |
|  | OE | **Enacted stigma** |
|  | RFT | **Unyanyapaa toka kwa wengine** |
|  | BTVI | **Stigma from others** |
|  | BTV2 | **Stigma from others** |
| 1 | OE | Family members have thought that I cannot be trusted |
|  | RFT | Wanafamilia wamefikiri kuwa siwezi kuaminika |
|  | BTVI | Family members have thought that I cannot be trusted |
|  | BTV2 | Family members have thought that I am not reliable |
| 2 | OE | Family members have looked down on me |
|  | RFT | Wanafamilia wamenidharau |
|  | BTVI | Family members have despised me |
|  | BTV2 | Family members have despised me |
| 3 | OE | Family members have treated me differently |
|  | RFT | Wanafamilia wamenichukulia/tendea tofauti |
|  | BTVI | Family members have treated me different |
|  | BTV2 | Family members have perceived/treated me differently |
| 4 | OE | Healthcare workers have not listened to my concerns |
|  | RFT | Watoa huduma za afya hawajasikiliza haja zangu |
|  | BTVI | Healthcare providers have not listened to my needs |
|  | BTV2 | Health care providers have not attended to my concerns. |
| 5 | OE | Healthcare workers have thought that I’m pill shopping, or trying to con them into giving me prescription medications to get high or sell |
|  | RFT | Watoa huduma za afya wamedhani nakusanya vidonge au najaribu kuwarubuni wanipatie cheti cha dawa ili nizitumie kupata raha au niuze |
|  | BTVI | Healthcare providers have thought that I am collecting pills or trying to lure them into giving me prescriptions so that I can use it for pleasure or selling |
|  | BTV2 | Health care providers have thought that I am collecting tablets or trying to incite them to prescribe tables for me so that I can use them to sedate myself or sell them |
| 6 | OE | Healthcare workers have given me poor care |
|  | RFT | Watoa huduma za afya wamenipa huduma duni |
|  | BTVI | Healthcare providers have given me poor care |
|  | BTV2 | Health care providers have given me poor services |
|  | OE | **Anticipated stigma** |
|  | RFT | **Unyanyapaa unaotarajiwa kutoka kwa wengine** |
|  | BTVI | **Expected stigma from others** |
|  | BTV2 | **Stigma expected from others** |
| 7 | OE | Family members will think that I cannot be trusted |
|  | RFT | Wanafamilia watafikiria siwezi kuaminiwa |
|  | BTVI | Family members will think that I cannot be trusted |
|  | BTV2 | Family members shall think that I am not reliable |
| 8 | OE | Family members will look down on me |
|  | RFT | Wanafamilia watanidharau |
|  | BTVI | Family members will despise me |
|  | BTV2 | Family members shall despise me |
| 9 | OE | Family members will treat me differently |
|  | RFT | Wanafamilia watanichukulia/tendea/ tofauti |
|  | BTVI | Family members will treat me differently |
|  | BTV2 | Family members shall perceive/treat me differently |
| 10 | OE | Healthcare workers will not listen to my concerns |
|  | RFT | Watoa huduma za afya hawatasikiliza haja zangu |
|  | BTVI | Healthcare providers will not listen to my needs |
|  | BTV2 | Health care providers shall never attend to my concerns |
| 11 | OE | Healthcare workers will think that I’m pill shopping, or trying to con them into giving me prescription medications to get high or sell |
|  | RFT | Watoa huduma za afya watadhani nakusanya vidonge au najaribu kuwarubuni wanipatie cheti cha dawa ili nizitumie nipate raha au niuze |
|  | BTVI | Healthcare providers will think that I am collecting pills or trying to lure them into giving me prescriptions so that I can use it for pleasure or selling |
|  | BTV2 | Health care providers shall think that I am collecting tablets or trying to incite them to prescribe tables for me so that I can use them to sedate myself or sell them |
| 12 | OE | Healthcare workers will give me poor care |
|  | RFT | Watoa huduma za afya watanipa huduma duni |
|  | BTVI | Healthcare providers will give me poor care |
|  | BTV2 | Health care providers shall give me poor services |
|  | OE | **Internalized stigma** |
|  | RFT | **Unyanyapaa binafsi** |
|  | BTVI | **Personal Stigma** |
|  | BTV2 | **Personal stigma** |
| 13 | OE | Having used alcohol/drugs makes me feel like I’m a bad person |
|  | RFT | Kutumia kwangu pombe/ dawa za kulevya kunanifanya nijihisi kuwa mtu mbaya |
|  | BTVI | My use of alcohol/drugs makes me feel like a bad person |
|  | BTV2 | Use of liquor/drugs makes me feel that I am a bad person |
| 14 | OE | I feel I’m not as good as others because I use alcohol/drugs |
|  | RFT | Najihisi sio mtu mzuri kama wengine kwasababu natumia pombe/ dawa za kulevya |
|  | BTVI | I feel like I am not a good person like everyone else because I use alcohol/drugs |
|  | BTV2 | I feel that I am not as good person as others because I take alcohol/use drugs |
| 15 | OE | I feel ashamed of having used alcohol/drugs |
|  | RFT | Najisikia aibu kwa kuwa nimetumia pombe/ dawa za kulevya |
|  | BTVI | I feel shame because I have use alcohol/drugs |
|  | BTV2 | I feel shy because I have taken liquor/used drugs |
| 16 | OE | I think less of myself because I used alcohol/drugs |
|  | RFT | Najiona sina thamani kwasababu nilitumia pombe/ dawa za kulevya |
|  | BTVI | I feel worthless because I have used alcohol/drugs |
|  | BTV2 | I feel devalued because I took liquor/used drugs |
| 17 | OE | Having used alcohol/drugs makes me feel unclean |
|  | RFT | Kutumia pombe/ dawa za kulevya kunafanya nijihisi sio msafi/mdhambi |
|  | BTVI | Using alcohol/drugs makes me feel dirty/sinful |
|  | BTV2 | Using liquor/drugs makes me feel unclean/sinful |
| 18 | OE | Having used alcohol/drugs is disgusting to me |
|  | RFT | Kutumia pombe/ dawa za kulevya ni kero kwangu |
|  | BTVI | Using alcohol/drugs is irritating to me |
|  | BTV2 | Using alcohol/drugs is boring to me |

1. **Perceived Stigma of Substance Abuse Scale (PSAS) - OE**

**Kipimo cha hofu ya kunyanyapaliwa kwa matumizi ya dawa za kulevya – RFT**

**Response options**

| OE | **Very strongly disagree** | **Strongly disagree** | **Disagree** | **Agree** | **Strongly agree** | **Very strongly agree** |
| --- | --- | --- | --- | --- | --- | --- |
| RFT | **Sikubali kabisa kabisa** | **Sikubali kabisa** | **Sikubali** | **Nakubali** | **Nakubali kabisa** | **Nakubali kabisa kabisa** |
| BTVI | **Vehemently Disagree** | **Strongly Disagree** | **Disagree** | **Agree** | **Strongly Agree** | **Vehemently Agree** |
| BTV2 | **Very Strongly disagree** | **Strongly disagree** | **Disagree** | **Agree** | **Strongly agree** | **Very strongly agree** |

**Items**

| **Sn** | OE | **Item** | |
| --- | --- | --- | --- |
|  | RFT | **Kipengele** | |
|  | BTVI | **Category** | |
|  | BTV2 | **Aspect** | |
| 1 | OE | Most people would willingly accept someone who has been treated for substance use as a close friend | |
|  | RFT | Watu wengi watamkubali kwa hiari mtu ambaye amepata tiba ya matumizi ya dawa za kulevya kama ad aw wa karibu | |
|  | BTVI | Most people will voluntarily accept a person who has received drug treatment as a close friend | |
|  | BTV2 | Most people shall willingly accept a person who has been treated for drug abuse as a close friend | |
| 2 | OE | Most people believe that someone who has been treated for substance use is just as trustworthy as the average citizen | |
|  | RFT | Watu wengi huamini kuwa mtu ambaye amepata tiba ya matumizi ya dawa za kulevya ni mwaminifu kama raia wa kawaida | |
|  | BTVI | Many people believe that a person who has been treated for drug abuse is as honest as the average citizen | |
|  | BTV2 | Most people believe that a person who has been treated for drug abuse is reliable just like a normal citizen | |
| 3 | OE | Most people would accept someone who has been treated for substance use as a teacher of young children in a public school | |
|  | RFT | Watu wengi watamkubali mtu ambaye ametibiwa kwa matumizi ya dawa za kulevya kuwa mwalimu wa ad aw wadogo kwenye shule ya uma | |
|  | BTVI | Most people will accept a person who has been treated for drug use as a teacher for young children in a public school | |
|  | BTV2 | Most people shall accept a person who has been treated for drugs as a teacher of their children in public schools | |
| 4 | OE | Most people would hire someone who has been treated for substance use to take care of their children | |
|  | RFT | Watu wengi watamuajiri mtu ambaye amepata tiba ya matumizi ad awa za kulevya kama muangalizi wa ad aw wao | |
|  | BTVI | Most people will hire someone who has received drug treatment as a baby sitter | |
|  | BTV2 | Most people shall employ a person who has been treated for drug abuse as a care taker of their children | |
| 5 | OE | Most people think less of a person who has been in treatment for substance use | |
|  | RFT | Watu wengi hawamthamini mtu ambaye amekwisha kuwa katika matibabu ya matumizi ad awa za kulevya | |
|  | BTVI | Most people do not appreciate someone who has been in drug treatment | |
|  | BTV2 | Most people do not value a person who has ever been under treatment for drug abuse. | |
| 6 | OE | Most employers will hire someone who has been treated for substance use if he or she is qualified for the job | |
|  | RFT | Waajiri wengi watamuajiri mtu aliyepata tiba ya matumizi ya dawa za kulevya kama atakidhi vigezo vya ajira | |
|  | BTVI | Most employers will hire someone who has received drug treatment if he or she meets the employment criteria | |
|  | BTV2 | Most employers shall employ a person who has been treated for drug abuse if he/she meets terms of employment | |
| 7 | OE | Most employers will pass over the application of someone who has been treated for substance use in favor of another applicant | |
|  | RFT | Waajiri wengi watatupilia mbali maombi ya ajira ya mtu aliyeekwishapata tiba ya matumizi ya dawa za kulevya kwa kumpendelea mwombaji mwingine | |
|  | BTVI | Most employers will reject a job application for someone who has already received drug treatment in favor of another applicant | |
|  | BTV2 | Most employers shall discard job  application from a person who has ever been treated for drug abuse in favour of another applicant | |
| 8 | OE | Most people would be willing to date someone who has been treated for substance use | |
|  | RFT | Watu wengi watakuwa tayari kuwa na mahusiano ya kimapenzi na mtu aliyepata tiba ya matumizi ya dawa za kulevya | |
|  | BTVI | Most people will be willing to have sexual relations with someone who has undergone drug treatment | |
|  | BTV2 | Most people shall be ready to have sexual relationships with a person who has been treated for drug abuse | |
|  | OE | For the Interviewer: After you asked the question, did the participant ask you to repeat asking any part of the question? | Yes. No  If yes, what part? |
|  | RFT | Kwa mdodosaji: Baada ya kuuliza swali, je mshiriki alikuomba urudie kuuliza kipengele chochote cha swali? | Ndio. Hapana  Kama ndio, sehemu gani? |
|  | BTVI | For Interviewer: After asking a question, did the participant ask you to repeat any aspect of the question? | Yes. No.  If yes, which? |
|  | BTV2 | For the researcher: after you asked the participant a question, did he/she ask you to repeat any part of the question? | Yes. No  If yes, which part? |
|  | OE | For the Interviewer: After you asked the question, did the participant requested for additional clarification of the question or qualify their answers? | Yes. No  If yes, what was the clarification? |
|  | RFT | Kwa mdodosaji: Baada ya kuuliza swali, je mshiriki aliomba ufafanuzi wa ziada juu ya swali hilo? | Ndio. Hapana  Kama ndio, ilikuwa ni ufafanuzi gani? |
|  | BTVI | For Interviewer: After asking the question, did the participant request additional clarification on the question? | Yes. No.  If yes, what clarification? |
|  | BTV2 | For the researcher: After you asked a question, did the participant request further clarification on the question? | Yes.No  If yes, what kind of clarification? |
|  | OE | c) Ask the participant the following questions and document what the participant says:  i) Can you tell me in your own words what this question was asking?? | |
|  | RFT | c) Ask the participant the following questions and write down the responses:  i) Can you explain to me in your own words what this question was about? | |
|  | BTVI | c) Ask the participant the following questions and write down what he or she said:  i) Can you explain to me in your own words what this question asked? | |
|  | BTV2 | c) Muulize mshiriki maswali  yafuatayo na uandike alichosema:  i) unaweza kunielezea kwa maneno yako mwenyewe swali hili liliuliza nini? | |
|  | OE | ii) did you have any difficulty using the response options. Why? | |
|  | RFT | ii) Ulipata ugumu wowote katika kutumia machaguo ya majibu? Kwanini? | |
|  | BTVI | ii) Did you find any difficulty in using the answer options? Why? | |
|  | BTV2 | ii) Did you face any difficulty choosing the answers? Why? | |
|  | OE | iii) how did you decide to answer this question?  (probe: what was your thought process to arrive at your response?) Use this probe if the participant did not understand what you meant. | |
|  | RFT | iii) Uliamua kulijibu kwa namna gani swali hili?  (dodosa: ulitafakari nini mpaka kufikia kwenye jibu lako?) Dodosa hivi endapo mshiriki hakuelewa ulichomaanisha | |
|  | BTVI | iii) How did you decide to answer this question?  (Probe: what did you think about until you got to your answer?) Probe like this if the participant did not understand what you meant. | |
|  | BTV2 | iii) How did you decide to answer this question?  (probe: what did you think to arrive to your answer?) probe this way if the participant did not understand what you meant | |
|  | OE | Was there a particular experience or experiences that you reflected upon before/while answering the question? | |
|  | RFT | Je, kulikua na hisia/mapito/uzoefu zozote / yoyote / wowote ulizozikumbuka / uliyoyakumbuka / ulioukumbuka kabla au wakati unajibu swali? | |
|  | BTVI | Were there any feelings/experiences/anything you remembered/remember/before or while answering the questions? | |
|  | BTV2 | Were there any feelings/past experiences you recalled before or when answering the question? | |
