# Supplementary material for: Translation and cultural adaptation of drug use stigma and HIV stigma measures among people who use drugs in Tanzania
Source: PLoS One. 2023 Oct 19;18(10):e0292642. doi: 10.1371/journal.pone.0292642 (PMC10586607; doi:10.1371/journal.pone.0292642)
Supplement: S2 File — (DOCX) [file pone.0292642.s002.docx]

**S2 File. Stigma measures for people who use drugs who are living with HIV**

**EXPERIENCED DRUG USE STIGMA**

We would now like to ask you about negative experiences you may have had interacting with people such as family, neighbors, friends, bosses, work colleagues, or peers because you use drugs. For the following questions please think about how often the following things happen to you because you use drugs. Please tell me whether the following experiences have never happened, happened once, a few times, or often.

| **Sn** | **Item** | **Never** | **Once** | **A few times** | **Often** |
| --- | --- | --- | --- | --- | --- |
| EDU1 | How often have family members avoided you because you use drugs? |  |  |  |  |
| EDU2 | How often have family members thought that you cannot be trusted because you use drugs? |  |  |  |  |
| EDU3 | How often have family members looked down on you because you use drugs? |  |  |  |  |
| EDU4 | How often have family members treated you differently because you use drugs? |  |  |  |  |
| EDU5 | How often have family members talked badly or gossiped about you because you use drugs? |  |  |  |  |
| EDU6 | How often have other people (not family members) talked badly or gossiped about you because you use drugs? |  |  |  |  |
| EDU7 | How often have peers (people who use drugs) talked badly or gossiped about you because you use drugs? |  |  |  |  |
| EDU8 | How often has someone verbally harassed you (e.g., yelled, scolded) because you use drugs? |  |  |  |  |
|  | HCSB1. Have you ever needed general health care that is not related to drugs use or HIV?  1= Yes  0= No Skip to EDU 16, **(Skip EDU9 – EDU15 and EH8 – EH12)**  HCSB2. Did you seek for health care from a health facility?  1= Yes  0= No **(Skip EDU9 – EDU15 and EH8 – EH12)**  HCSB3. To which health facility did yo ugo to seek for health care?  1= CTC **(Skip EDU9 – EDU15 and EH8 – EH12)**  2= General health facility  3= Any other (Mention) ________________________________  I am now going to ask you some questions about your negative experiences when you have gone to a clinic or hospital for general healthcare, that is for help with other health needs **that ARE NOT** related to **drug use or HIV treatment,** for example, general health care needs can include health needs like malaria, flu, or an accident because you use drugs. Please tell me whether the following experiences have never happened, happened once, a few times, or often. | | | | |
| EDU9 | How often have healthcare workers not listened to your concerns when you have gone to a health facility for general health care because you use drugs? |  |  |  |  |
| EDU10 | How often have healthcare workers avoided physical contact with you when you have gone to a health facility for general health care because you use drugs? |  |  |  |  |
| EDU11 | How often have healthcare workers disrespected you when you have gone to a health facility for general health care because you use drugs? |  |  |  |  |
| EDU12 | How often have healthcare workers thought that you are pill shopping, or trying to con them into giving you prescription medications to get high or sell when you have gone to a health facility for general health care because you use drugs? |  |  |  |  |
| EDU13 | How often have healthcare workers given you poor care when you have gone to a health facility for general health care because you use drugs? |  |  |  |  |
| EDU14 | How often have health workers verbally harassed you (e.g., yelled, scolded) when you have gone to a health facility for general health care because you use drugs? |  |  |  |  |
| **ONLY FOR PLHIV RESPONDENTS WHO ARE ON ANTIRETROVIRAL TREATMENT:**  **Thank you for telling me that you are receiving antiretroviral treatment. I am now going to ask you about your experiences when you go for HIV treatment at the CTC.** Please tell me whether the following experiences have never happened, happened once, a few times, or often. | | | | | |
| EDU16 | How often have healthcare workers not listened to your concerns when you have gone for HIV care at the CTC because you use drugs? |  |  |  |  |
| EDU17 | How often have healthcare workers avoided physical contact with you when you have gone for HIV care at the CTC because you use drugs? |  |  |  |  |
| EDU18 | How often have healthcare workers disrespected you when you have gone for HIV care at the CTC because you use drugs? |  |  |  |  |
| EDU19 | How often have health workers verbally harassed you (e.g., yelled, scolded) when you have gone for HIV care at the CTC because you use drugs? |  |  |  |  |
| EDU20 | How often have healthcare workers made you wait longer than other clients when you have gone for HIV care at the CTC because you use drugs? |  |  |  |  |
| EDU21 | How often have healthcare workers have given you priority and treated you first instead of other clients who were waiting for health services before you when you have gone for HIV care at the CTC because you use drugs? |  |  |  |  |
| EDU22 | How often have healthcare workers thought that you are pill shopping, or trying to con them into giving you prescription medications to get high or sell when you have gone **for HIV care** at the CTC because you use drugs? |  |  |  |  |
| EDU23 | How often have healthcare workers given you poor care when you have gone for HIV care at the CTC because you use drugs? |  |  |  |  |

**ANTICIPATED DRUG USE STIGMA**

In the previous section, you shared about negative experiences you have had, because you use drugs. Sometimes, because of our previous negative experiences, or because we see other people being treated negatively, we may have worry or concerns about how we may be treated by other people. In the following section I will ask you a series of statements about worries or concerns you might have about what other people think or may do, because you use drugs. **Please tell me whether the following experiences have never happened, happened once, a few times, or often.**

| **Sn** | **Item** | **Never** | **Once** | **A few times** | **Often** |  |
| --- | --- | --- | --- | --- | --- | --- |
| ADU1 | How often do you worry that family members will avoid you because you use drugs? |  |  |  |  |  |
| ADU2 | How often do you worry that family members will think that you cannot be trusted because you use drugs? |  |  |  |  |  |
| ADU3 | How often do you worry that family members will look down on you because you use drugs? |  |  |  |  |  |
| ADU4 | How often do you worry that family members will treat you differently because you use drugs? |  |  |  |  |  |
| ADU5 | How often do you worry that your peers/other people who use or used drugs will avoid you because you use drugs? |  |  |  |  |  |
| ADU6 | How often do you worry your peers/other people who use or used drugs will gossip about you because you use drugs? |  |  |  |  |  |
| ADU7 | How often do you worry that your peers/other people who use/used drugs will reject you because you use drugs? |  |  |  |  |  |
| ADU8 | How often do you worry that people who know you use drugs will tell others? |  |  |  |  |  |
| ADU9 | How often do you worry that people may judge you when they learn you use drugs? |  |  |  |  |  |
| I am now going to ask you about worries you may have when you go to a clinic or hospital for general healthcare. By general healthcare, I mean when you have gone to a health facility for health needs like like malaria, flu, an accident **that ARE NOT** related to **drug use or HIV treatment**. **Please tell me whether the following experiences have never happened, happened once, a few times, or often.** | | | | | | |
| ADU10 | How often do you worry that healthcare workers will not listen to your concerns when you go to a health facility for general health care because you use drugs? |  |  |  |  |  |
| ADU11 | How often do you worry that healthcare workers will think that you are pill shopping, or trying to con them into giving me prescription medications to get high or sell when you go to a health facility for general care because you use drugs? |  |  |  |  |  |
| ADU12 | How often do you worry that healthcare workers will give you poor care when you go to a health facility for general health care because you use drugs? |  |  |  |  |  |
| ADU13 | How often do you worry that you will be made to wait longer than other clients when you go to a health facility for general health care because you use drugs? |  |  |  |  |  |
| ADU14 | How often do you worry that you will be disrespected when you go to a health facility for general health care because you use drugs? |  |  |  |  |  |
| ADU15 | How often do you worry that you will be judged when you go to a health facility for general health care because you use drugs? |  |  |  |  |  |
| ADU16 | How often do you worry that you will be shamed when you go to a health facility for general health care because you use drugs? |  |  |  |  |  |
| ADU17 | How often do you worry that you will be blamed when you go to a health facility for general health care because you use drugs? |  |  |  |  |  |
| ADU18 | How often do you worry that you will be ignored when you go to a health facility for general health care because you use drugs? |  |  |  |  |  |
| ADU19 | How often do you worry that health workers will think that you are going to steal from them when you go to a health facility for general health care because you use drugs? |  |  |  |  |  |
| ADU20 | How often do you worry that health care workers will disclose to others that you use drugs when you go to a health facility for general health care? |  |  |  |  |  |
| ADU21 | How often do you worry that you will not be given care if you do not dress properly when you go to a health facility for general health care because you use drugs? |  |  |  |  |  |
| ADU22 | How often do you worry that you will be verbally harassed (e.g., yell, scold) when you go to a health facility for general health care because you use drugs? |  |  |  |  |  |
| **ONLY FOR PLHIV RESPONDENTS WHO ARE ON ART:**  **Thank you for sharing with me that you are currently taking HIV treatment (ART). Sometimes, people have worries or concerns around going for treatment. I am now going to ask you about your concerns or worries around going for HIV treatment. For each question, please tell me if you have never had worry, or had worry once, a few times, or often.** | | | | | | |
| ADU23 | How often do you worry that healthcare workers will not listen to your concerns when you go to CTC for HIV care because you use drugs? |  |  |  |  |  |
| ADU24 | How often do you worry that healthcare workers will avoid physical contact with you when you go for HIV care at the CTC because you use drugs? |  |  |  |  |  |
| ADU25 | How often do you worry that you will be disrespected when you go for HIV care at the CTC because you use drugs? |  |  |  |  |  |
| ADU26 | How often do you worry that healthcare workers will give you poor care when you go for HIV care at the CTC because you use drugs? |  |  |  |  |  |
| ADU27 | How often do you worry that you will be made to wait longer than other clients when you go for HIV care at the CTC because you use drugs? |  |  |  |  |  |
| ADU28 | How often do you worry that you will be judged when you go for HIV care at the CTC because you use drugs? |  |  |  |  |  |
| ADU29 | How often do you worry that you will be shamed when you go for HIV care at the CTC because you use drugs? |  |  |  |  |  |
| ADU30 | How often do you worry that you will be blamed when you go for HIV care at the CTC because you use drugs? |  |  |  |  |  |
| ADU31 | How often do you worry that you will be ignored when you go for HIV care at the CTC because you use drugs? |  |  |  |  |  |
| ADU32 | How often do you worry that health workers will think that you are going to steal from them when you go HIV care at the CTC because you use drugs? |  |  |  |  |  |
| ADU33 | How often do you worry that health care workers will disclose to others that you use drugs when you go for HIV care at the CTC? |  |  |  |  |  |
| ADU34 | How often do you worry that you will be verbally harassed (e.g., yelled, scolded) when you go for HIV care at the CTC because you use drugs? |  |  |  |  |  |
| ADU35 | How often do you worry that you will not be given care if you do not dress properly when you go for HIV care at the CTC because you use drugs? |  |  |  |  |  |
| **ONLY FOR PLHIV RESPONDENTS WHO ARE NOT ON ART:**  **Thank you for sharing with me that you are not currently taking HIV treatment (ART). Sometimes, people may have concerns or worries about going to CTC, about what might happen at the CTC. For the next series of questions, I am going to ask you whether you have concerns or worries related to *if you were to go for HIV treatment*. For each question, please tell me if you have never had worry, or had worry once, a few times, or often.** | | | | | | |
| ADU36 | How often do you worry that healthcare workers will not listen to your concerns if you were to go to CTC for HIV care because you use drugs? |  |  |  |  |  |
| ADU37 | How often do you worry that healthcare workers will avoid physical contact with you if you were to go for HIV care at the CTC because you use drugs? |  |  |  |  |  |
| ADU38 | How often do you worry that you will be disrespected if you were to go for HIV care at the CTC because you use drugs? |  |  |  |  |  |
| ADU39 | How often do you worry that healthcare workers will give you poor care if you were to go for HIV care at the CTC because you use drugs? |  |  |  |  |  |
| ADU40 | How often do you worry that you will be made to wait longer than other clients if you were to go for HIV care at the CTC because you use drugs? |  |  |  |  |  |
| ADU41 | How often do you worry that you will be judged if you were to go for HIV care at the CTC because you use drugs? |  |  |  |  |  |
| ADU42 | How often do you worry that you will be shamed if you were to go for HIV care at the CTC because you use drugs? |  |  |  |  |  |
| ADU43 | How often do you worry that you will be blamed if you were to go for HIV care at the CTC because you use drugs? |  |  |  |  |  |
| ADU44 | How often do you worry that you will be ignored if you were to go for HIV care at the CTC because you use drugs? |  |  |  |  |  |
| ADU45 | How often do you worry that health workers will think that you are going to steal from them if you were to go for HIV care at the CTC because you use drugs? |  |  |  |  |  |
| ADU46 | How often do you worry that health care workers will disclose to others that you use drugs if you were to go for HIV care at the CTC? |  |  |  |  |  |
| ADU47 | How often do you worry that you will be verbally harassed (e.g., yelled, scolded) if you were to go for HIV care at the CTC because you use drugs? |  |  |  |  |  |
| ADU48 | How often do you worry that you will not be given care if you do not dress properly if you were to go for HIV care at the CTC because you use drugs? |  |  |  |  |  |

**PERCEIVED DRUG USE STIGMA**

I have asked you about your negative experiences with other people, as well as worries you have about how other people might think about or treat you, because you use drugs. Now I want to ask you about what you think other people (the general population) think or assume about people who use drugs. The following section includes a series of statements that represent assumptions or views other people (people in the general population/ community) might have about people who use drugs. To answer these questions, please consider the thoughts, ideas, and impressions the general population has about people who use drugs. Please rate the extent to which you agree or disagree **that other people (people in the general population)** believe these statements, not the extent to which you personally believe these statements. Please tell me if you strongly disagree, disagree, neither disagree or agree, agree, or strongly agree that other people believe the following statements.

|  | **Item** | **Strongly disagree** | **Disagree** | **Neither disagree or agree** | **Agree** | **Strongly agree** |
| --- | --- | --- | --- | --- | --- | --- |
| PDU1 | Most people believe that a person who uses drugs is dirty |  |  |  |  |  |
| PDU2 | Most people who use drugs are rejected when others find out that they use drugs |  |  |  |  |  |
| PDU3 | People who use drugs lose their jobs when their employers find out they use drugs |  |  |  |  |  |
| PDU4 | Most people think that a person who uses drugs is disgusting |  |  |  |  |  |
| PDU5 | Most people judge people who use drugs |  |  |  |  |  |
| PDU6 | Most people do not respect people who use drugs |  |  |  |  |  |
| PDU7 | Most people think people who use drugs have only themselves to blame for their drug use |  |  |  |  |  |
| PDU8 | Most people verbally harass (e.g., yell, scold) people who use drugs |  |  |  |  |  |
| PDU9 | Most people would willingly accept someone who has been treated for drug use as a close friend (not a sexual partner) |  |  |  |  |  |
| PDU10 | Most people believe that someone who has been treated for drug use is just as trustworthy as the average citizen |  |  |  |  |  |
| PDU11 | Most people would accept someone who has been treated for drug use as a teacher of children in a primary school |  |  |  |  |  |
| PDU12 | Most people would hire someone who has been treated for drug use to take care of their children |  |  |  |  |  |
| PDU13 | Most people think less of a person who has been in treatment for drug use |  |  |  |  |  |
| PDU14 | Most employers will hire someone who has been treated for drug use if he or she is qualified for the job |  |  |  |  |  |
| PDU15 | Most employers will pass over the application of someone who has been treated for drug use in favor of another applicant |  |  |  |  |  |
| PDU16 | Most people would be willing to marry someone who has been treated for drug use |  |  |  |  |  |

**INTERNALIZED DRUG USE STIGMA**

**The next set of questions are about feelings and thoughts you may have experienced because you use drugs. Please rate the extent to which you agree or disagree with each of the following statements. Please select the answer that best reﬂects your thoughts and feelings today.** Please tell me if you strongly disagree, disagree, neither disagree or agree, agree, or strongly agree with the following statements.

| **Sn** | **Item** | **Strongly disagree** | **Disagree** | **Neither disagree or agree** | **Agree** | **Strongly agree** |
| --- | --- | --- | --- | --- | --- | --- |
| IDU1 | It is difficult to tell people about being a person who uses drugs |  |  |  |  |  |
| 1DU2 | Using drugs makes me feel dirty |  |  |  |  |  |
| 1DU3 | I feel guilty that I use drugs |  |  |  |  |  |
| 1DU4 | I am ashamed that I use drugs |  |  |  |  |  |
| 1DU5 | I sometimes feel worthless because I use drugs |  |  |  |  |  |
| 1DU6 | I hide my status of using drugs from others |  |  |  |  |  |

**EXPERIENCED HIV STIGMA**

We would now like to ask you about negative experiences you may have had interacting with people such as family, neighbors, friends, bosses, work colleagues, or peers because you are living with HIV. For the following questions please think about how often the following things happen to you because you are a person living with HIV. **Please tell me whether the following experiences have never happened, happened once, a few times, or often.**

| Sn | **Statement** | **Never** | | **Once** | | **A few times** | | **Often** | |
| --- | --- | --- | --- | --- | --- | --- | --- | --- | --- |
| EH1 | How often have family members avoided you because you are living with HIV |  | |  | |  | |  | |
| EH2 | How often have family members looked down on you because you are living with HIV |  | |  | |  | |  | |
| EH3 | How often have family members treated you differently because you are living with HIV |  | |  | |  | |  | |
| EH4 | How often have family members talked badly or gossiped about you because of your HIV status? |  | |  | |  | |  | |
| EH5 | How often have other people (not family members) talked badly gossiped about you because of your HIV status? |  | |  | |  | |  | |
| EH6 | How often have peers (people who use or used drugs) talked badly or gossiped about you because of your HIV status? |  | |  | |  | |  | |
| EH7 | How often has someone verbally harassed you (e.g., yell, scold) because of your HIV status? |  | |  | |  | |  | |
| Now I am going to ask you about your experiences when you have gone to a health facility for general care for health needs like like malaria, flu that **are not** related to drug use or HIV treatment. **Please tell me whether the following experiences have never happened, happened once, a few times, or often.** | | | | | | | | | |
| EH8 | How often have healthcare workers not listened to your concerns when you have gone to a health facility for general care because you are living with HIV? | |  | |  | |  | |  |
| EH9 | How often have healthcare workers avoided physical contact with you when you have gone to a health facility for general health care because you are living with HIV? | |  | |  | |  | |  |
| EH10 | How often have healthcare workers treated you disrespectfully when you have gone to a health facility for general health care because you are living with HIV? | |  | |  | |  | |  |
| EH11 | How often have healthcare workers verbally harassed you (e.g., yelled, scolded) when you have gone to a health facility for general health care because you are living with HIV? | |  | |  | |  | |  |

**ANTICIPATED HIV STIGMA**

In the previous section, you shared about negative experiences you have had, because you are a person living with HIV. Sometimes, because of our previous negative experiences, or because we see other people being treated negatively, we may have worry or concerns about how we may be treated by other people. In the following section I will ask you a series of statements about worries or concerns you might have about what other people think or may do, because you are living with HIV. **Please tell me whether the following experiences have never happened, happened once, a few times, or often.**

| **Sn** | **Statement** | **Never** | **Once** | **A few times** | **Often** |
| --- | --- | --- | --- | --- | --- |
| AH1 | How often do you worry that family members will avoid you because you are living with HIV? |  |  |  |  |
| AH2 | How often do you worry that family members will think that you cannot be trusted because you are living with HIV? |  |  |  |  |
| AH3 | How often do you worry that family members will look down on you because you are living with HIV? |  |  |  |  |
| AH4 | How often do you worry that family members will treat you differently because you are living with HIV? |  |  |  |  |
| AH5 | How often do you worry that your peers/other people who use or used drugs will avoid you because you are living with HIV? |  |  |  |  |
| AH6 | How often do you worry your peers/other people who use or used drugs will gossip about you because you are living with HIV? |  |  |  |  |
| AH7 | How often do you worry that your peers/other people who use/used drugs will reject you because you are living with HIV? |  |  |  |  |
| AH8 | How often do you worry that people who know you have HIV will tell others? |  |  |  |  |
| AH9 | How often do you worry that people may judge you when they learn you have HIV? |  |  |  |  |
| Now I am going to ask you about worries you may have when you go to a health facility for general care. By general care, I mean when you have gone to a health facility for health needs like like malaria, flu, an accident that **are not** related to HIV treatment. I want to understand your worries about going for general health care, because you are living with HIV. | | | | | |
| AH10 | How often do you worry that healthcare workers will not listen to your concerns when you go to a health facility for general health care because you are living with HIV? |  |  |  |  |
| AH11 | How often do you worry that healthcare workers will give you poor care when you go to a health facility for general health care because you are living with HIV? |  |  |  |  |
| AH12 | How often do you worry that you will be made to wait longer than other clients when you go to a health facility for general health care because you are living with HIV? |  |  |  |  |
| AH13 | How often do you worry that healthcare workers will avoid physical contact with you when you go to a health facility for general health care because you are living with HIV? |  |  |  |  |
| AH14 | How often do you worry you will be disrespected when you go to a health facility for general health care (, because you are living with HIV? |  |  |  |  |
| AH15 | How often do you worry that you will be judged when you go to a health facility for general health care because you are living with HIV? |  |  |  |  |
| AH16 | How often do you worry that you will be shamed when you go to a health facility for general health care because you are living with HIV? |  |  |  |  |
| AH17 | How often do you worry that you will be blamed when you go to a health facility for general health care because you are living with HIV? |  |  |  |  |
| AH18 | How often do you worry that you will be ignored when you go to a health facility for general health care (because you are living with HIV? |  |  |  |  |
| AH19 | How often do you worry that health care workers will disclose to others that you are living with HIV when you go to a health facility for general health care? |  |  |  |  |
| AH20 | How often do you worry that you will be verbally harassed (e.g., yelled ag, scolded) when you go to the health facility for general health care, because you are living with HIV |  |  |  |  |

**PERCEIVED HIV STIGMA**

The following section includes a series of statements that represent assumptions or views other people (people in the community) might have about persons who are living with HIV. To answer these questions, please consider the thoughts, ideas, and impressions the general population has about people who are living with HIV. Please rate the extent to which you agree or disagree that **other people believe these statements**, not the extent to which you personally believe these statements. Please tell me if you strongly disagree, disagree, neither disagree or agree, agree, or strongly agree that other people believe the following statements.

|  |  | **Strongly disagree** | **Disagree** | **Neither disagree or agree** | **Agree** | **Strongly agree** |
| --- | --- | --- | --- | --- | --- | --- |
| PH1 | Most people believe that a person who has HIV is dirty |  |  |  |  |  |
| PH2 | Most people with HIV are rejected when others find out that they are living with HIV |  |  |  |  |  |
| PH3 | People with HIV lose their jobs when their employers find out they are living with HIV |  |  |  |  |  |
| PH4 | Most people think that a person with HIV is disgusting |  |  |  |  |  |
| PH5 | Most people judge people who are HIV positive |  |  |  |  |  |
| PH6 | Most people do not respect people who are HIV positive |  |  |  |  |  |
| PH7 | Most people think people living with HIV have only themselves to blame for being HIV positive |  |  |  |  |  |
| PH8 | Most people think people who are HIV positive are promiscuous |  |  |  |  |  |
| PH9 | Most people verbally harass (e.g., yell, scold) someone who is living with HIV |  |  |  |  |  |
| PH10 | Most people would willingly accept someone who is living with HIV as a close friend (not a sexual partner) |  |  |  |  |  |
| PH11 | Most people would accept someone who is living with HIV as a teacher of children in a primary school |  |  |  |  |  |
| PH12 | Most people would hire someone who is living with HIV to take care of their children |  |  |  |  |  |
| PH13 | Most people think less of a person who is living with HIV |  |  |  |  |  |
| PH14 | Most employers will hire someone who is living with HIV if he or she is qualified for the job |  |  |  |  |  |
| PH15 | Most employers will pass over the application of someone who is living with HIV in favor of another applicant |  |  |  |  |  |
| PH16 | Most people would be willing to marry someone who is living with HIV |  |  |  |  |  |

**INTERNALIZED HIV STIGMA**

The next set of questions are about feelings and thoughts you may have experienced because you are living with HIV. Please rate the extent to which you agree or disagree with each of the following statements. Please select the answer that best reﬂects your thoughts and feelings today. Please tell me if you strongly disagree, disagree, neither disagree or agree, agree, or strongly agree with the following statements.

| **Sn** | **Item** | **Strongly disagree** | **Disagree** | **Neither disagree or agree** | **Agree** | **Strongly agree** |
| --- | --- | --- | --- | --- | --- | --- |
| IH1 | It is difficult to tell people that I am HIV positive |  |  |  |  |  |
| IH2 | Being HIV positive makes me feel dirty |  |  |  |  |  |
| IH3 | I feel guilty that I am HIV positive |  |  |  |  |  |
| IH4 | I am ashamed that I am HIV positive |  |  |  |  |  |
| IH5 | I sometimes feel worthless because I am HIV positive |  |  |  |  |  |
| IH6 | I hide my HIV status from others |  |  |  |  |  |

## 
