# Supplementary material for: Translation and cultural adaptation of drug use stigma and HIV stigma measures among people who use drugs in Tanzania
Source: PLoS One. 2023 Oct 19;18(10):e0292642. doi: 10.1371/journal.pone.0292642 (PMC10586607; doi:10.1371/journal.pone.0292642)
Supplement: S5 File — (DOCX) [file pone.0292642.s005.docx]

**S5 File. Stigma measures for people who are on medically assisted treatment who are not living with HIV**

**EXPERIENCED DRUG USE STIGMA**

We would now like to ask you about negative experiences you may have had interacting with people such as family, neighbors, friends, bosses, work colleagues, or peers because you are on MAT. For the following questions please think about how often the following things happen to you because you are on MAT. Please tell me whether the following experiences have never happened, happened once, a few times, or often.

| **Sn** | **Item** | **Never (0)** | **Once (1)** | **A few times (2)** | **Often (3)** |
| --- | --- | --- | --- | --- | --- |
| EDUM1 | How often have family members avoided you because you are on MAT? |  |  |  |  |
| EDUM2 | How often have family members thought that you cannot be trusted because you are on MAT? |  |  |  |  |
| EDUM3 | How often have family members looked down on you because you use drugs? |  |  |  |  |
| EDUM4 | How often have family members treated you differently because you are on MAT? |  |  |  |  |
| EDUM5 | How often have family members talked badly or gossiped about you because you are on MAT? |  |  |  |  |
| EDUM6 | How often have other people (not family members) talked badly or gossiped about you because you use drugs? |  |  |  |  |
| EDUM7 | How often have peers (people who use drugs) talked badly or gossiped about you because you are on MAT? |  |  |  |  |
| EDUM8 | How often has someone verbally harassed you (e.g., yelled, scolded) because you are on MAT? |  |  |  |  |
|  | HCSB1. Have you ever needed general health care that is not related to drugs use or HIV?  1= Yes  0= No Skip to EDUM16, **(Skip EDUM9 – EDUM15 and EHM8 – EHM12)**  HCSB2. Did you seek for health care from a health facility?  1= Yes  0= No **(Skip EDUM9 – EDUM15 and EHM8 – EHM12)**  HCSB3. To which health facility did yo ugo to seek for health care?  1= CTC **(Skip EDUM9 – EDUM15 and EHM8 – EHM12)**  2= General health facility  3= Any other (Mention) ________________________________  I am now going to ask you some questions about your negative experiences when you have gone to a clinic or hospital for general healthcare, that is for help with other health needs **that ARE NOT** related to **drug use or HIV treatment,** for example, general health care needs can include health needs like malaria, flu, or an accident because you use drugs. Please tell me whether the following experiences have never happened, happened once, a few times, or often. | | | | |
| EDUM9 | How often have healthcare workers not listened to your concerns when you have gone to a health facility for general health care because you are on MAT? |  |  |  |  |
| EDUM10 | How often have healthcare workers avoided physical contact with you when you have gone to a health facility for general health care because you are on MAT? |  |  |  |  |
| EDUM11 | How often have healthcare workers disrespected you when you have gone to a health facility for general health care because you are on MAT? |  |  |  |  |
| EDUM12 | How often have healthcare workers thought that you are pill shopping, or trying to con them into giving you prescription medications to get high or sell when you have gone to a health facility for general health care because you are on MAT? |  |  |  |  |
| EDUM13 | How often have healthcare workers given you poor care when you have gone to a health facility for general health care because you are on MAT? |  |  |  |  |
| EDUM14 | How often have health workers verbally harassed you (e.g., yelled, scolded) when you have gone to a health facility for general health care because you are on MAT? |  |  |  |  |
| I am now going to ask you some questions about your negative experiences when you have gone to a MAT clinic. Please tell me whether the following experiences have never happened, happened once, a few times, or often. | | | | | |
| EDUM16 | How often have health workers verbally harassed you (e.g., yelled, scolded) when you have gone to a MAT clinic because you are on MAT? |  |  |  |  |
| EDUM17 | How often have healthcare workers not listened to your concerns when you have gone to a MAT clinic because you are on MAT? |  |  |  |  |
| EDUM18 | How often have healthcare workers thought that you are pill shopping, or trying to con them into giving you prescription medications to get high or sell when you have gone to a MAT clinic because you are on MAT? |  |  |  |  |
| EDUM19 | How often have healthcare workers given you poor care when you have gone to a MAT clinic because you are on MAT? |  |  |  |  |

**ANTICIPATED DRUG USE STIGMA**

In the previous section, you shared about negative experiences you have had, because you are on MAT. Sometimes, because of our previous negative experiences, or because we see other people being treated negatively, we may have worry or concerns about how we may be treated by other people. In the following section I will ask you a series of statements about worries or concerns you might have about what other people think or may do, because you are on MAT. **Please tell me whether the following experiences have never happened, happened once, a few times, or often.**

| **Sn** | **Item** | **Never (0)** | **Once (1)** | **A few times (2)** | **Often (3)** |  |
| --- | --- | --- | --- | --- | --- | --- |
| ADUM1 | How often do you worry that family members will avoid you because you are on MAT? |  |  |  |  |  |
| ADUM2 | How often do you worry that family members will think that you cannot be trusted because you are on MAT? |  |  |  |  |  |
| ADUM3 | How often do you worry that family members will look down on you because you are on MAT? |  |  |  |  |  |
| ADUM4 | How often do you worry that family members will treat you differently because you are on MAT? |  |  |  |  |  |
| ADUM5 | How often do you worry that your peers/other people who use or used drugs will avoid you because you are on MAT? |  |  |  |  |  |
| ADUM6 | How often do you worry your peers/other people who use or used drugs will gossip about you because you are on MAT? |  |  |  |  |  |
| ADUM7 | How often do you worry that your peers/other people who use/used drugs will reject you because you are on MAT? |  |  |  |  |  |
| ADUM8 | How often do you worry that people who know you are on MAT will tell others? |  |  |  |  |  |
| ADUM9 | How often do you worry that people may judge you when they learn you are on MAT? |  |  |  |  |  |
| I am now going to ask you about worries you may have when you go to a clinic or hospital for general healthcare needs like like malaria, flu, an accident **that ARE NOT** related to **drug use or HIV treatment**. **Please tell me whether the following experiences have never happened, happened once, a few times, or often.** | | | | | | |
| ADUM10 | How often do you worry that healthcare workers will not listen to your concerns when you go to a health facility for general health care because you are on MAT? |  |  |  |  |  |
| ADUM11 | How often do you worry that healthcare workers will think that you are pill shopping, or trying to con them into giving me prescription medications to get high or sell when you go to a health facility for general care because you are on MAT? |  |  |  |  |  |
| ADUM12 | How often do you worry that healthcare workers will give you poor care when you go to a health facility for general health care because you are on MAT? |  |  |  |  |  |
| ADUM13 | How often do you worry that you will be made to wait longer than other clients when you go to a health facility for general health care because you are on MAT? |  |  |  |  |  |
| ADUM14 | How often do you worry that you will be disrespected when you go to a health facility for general health care because you are on MAT? |  |  |  |  |  |
| ADUM15 | How often do you worry that you will be judged when you go to a health facility for general health care because you are on MAT? |  |  |  |  |  |
| ADUM16 | How often do you worry that you will be shamed when you go to a health facility for general health care because you are on MAT? |  |  |  |  |  |
| ADUM17 | How often do you worry that you will be blamed when you go to a health facility for general health care because you are on MAT? |  |  |  |  |  |
| ADUM18 | How often do you worry that you will be ignored when you go to a health facility for general health care because you are on MAT? |  |  |  |  |  |
| ADUM19 | How often do you worry that health workers will think that you are going to steal from them when you go to a health facility for general health care because you are on MAT? |  |  |  |  |  |
| ADUM20 | How often do you worry that health care workers will disclose to others that you are on MAT when you go to a health facility for general health care? |  |  |  |  |  |
| ADUM21 | How often do you worry that you will not be given care if you do not dress properly when you go to a health facility for general health care because you are on MAT? |  |  |  |  |  |
| ADUM22 | How often do you worry that you will be verbally harassed (e.g., yell, scold) when you go to a health facility for general health care because you are on MAT? |  |  |  |  |  |
| **I am going to ask you whether you have concerns or worries when you go to the MAT. For each question, please tell me if you have never had worry, or had worry once, a few times, or often.** | | | | | |  |
| ADUM49 | How often do you worry that health care workers at the MAT clinic will verbally harass (e.g., yelled, scolded) when you go to the MAT clinic because you are on MAT? |  |  |  |  |  |
| ADUM50 | How often do you worry that healthcare workers at the MAT clinic will not listen to your concerns when you go to the MAT clinic because you are on MAT? |  |  |  |  |  |
| ADUM51 | How often do you worry that healthcare workers at the MAT clinic will give you poor care when you go to the MAT clinic because you are on MAT? |  |  |  |  |  |
| ADUM52 | How often do you worry that health care workers at the MAT clinic will disrespect you when you go to the MAT clinic because you are on MAT? |  |  |  |  |  |
| ADUM53 | How often do you worry that you will be judged when you go to the MAT clinic because you are on MAT? |  |  |  |  |  |
| ADUM54 | How often do you worry that you will be shamed when you go to the MAT clinic because you are on MAT? |  |  |  |  |  |
| ADUM55 | How often do you worry that you will be blamed when you go to the MAT clinic because you are on MAT? |  |  |  |  |  |
| ADUM56 | How often do you worry that you will be ignored when you go to the MAT clinic because you are on MAT? |  |  |  |  |  |
| ADUM57 | How often do you worry that health workers at the MAT clinic will think that you are going to steal from them when you go to the MAT clinic because you are on MAT? |  |  |  |  |  |
| ADUM59 | How often do you worry that you will not be given care if you do not dress properly when you go to the MAT clinic because you are on MAT? |  |  |  |  |  |
| **ONLY FOR RESPONDENTS WHO ARE ON MAT WHO USE MAT TOGETHER WITH OTHER DRUGS**  **I am now going to ask you about your concerns or worries around going for treatment at the MAT clinic. For each question, please tell me if you have never had worry, or had worry once, a few times, or often.** | | | | | |  |
| ADUM60 | How often do you worry that health care workers at the MAT clinic will verbally harass (e.g., yelled, scolded) when you go to the MAT clinic because you are on MAT and you also use drugs? |  |  |  |  |  |
| ADUM61 | How often do you worry that healthcare workers at the MAT clinic will not listen to your concerns when you go to the MAT clinic because you are on MAT and you also use drugs? |  |  |  |  |  |
| ADUM62 | How often do you worry that healthcare workers at the MAT clinic will give you poor care when you go to the MAT clinic because you are on MAT and you also use drugs? |  |  |  |  |  |
| ADUM63 | How often do you worry that health care workers at the MAT clinic will disrespect you when you go to the MAT clinic because you are on MAT and you also use drugs? |  |  |  |  |  |
| ADUM64 | How often do you worry that you will be judged when you go to the MAT clinic because you are on MAT and you also use drugs? |  |  |  |  |  |
| ADUM65 | How often do you worry that you will be shamed when you go to the MAT clinic because you are on MAT and you also use drugs? |  |  |  |  |  |
| ADUM66 | How often do you worry that you will be blamed when you go to the MAT clinic because you are on MAT and you also use drugs? |  |  |  |  |  |
| ADUM67 | How often do you worry that you will be ignored when you go to the MAT clinic because you are on MA and you also use drugs T? |  |  |  |  |  |
| ADUM68 | How often do you worry that health workers at the MAT clinic will think that you are going to steal from them when you go to the MAT clinic because you are on MAT and you also use drugs? |  |  |  |  |  |
| ADUM69 | How often do you worry that you will not be given care if you do not dress properly when you go to the MAT clinic because you are on MAT and you also use drugs? |  |  |  |  |  |

In the following section I am now going to ask you about worries you might have if you were to go for ART if you were living with HIV because you are on MAT. **Please tell me whether the following experiences have never happened, happened once, a few times, or often.**

|  | Item | **Never (0)** | **Once (1)** | **A few times (2)** | **Often (3)** |
| --- | --- | --- | --- | --- | --- |
| ADUMAT49 | If you were living with HIV, how often do you think you would worry that healthcare workers would not listen to your concerns if you went to the HIV care and treatment center (CTC), because you are on MAT? |  |  |  |  |
| ADUMAT50 | If you were living with HIV, how often do you think you would worry that healthcare workers would avoid physical contact with you when you we went to the CTC, because you are on MAT? |  |  |  |  |
| ADUMAT51 | If you were living with HIV, how often do you think you would worry that healthcare workers would treat you disrespectful if you went to the CTC, because you are on MAT? |  |  |  |  |
| ADUMAT52 | If you were living with HIV, how often do you think you would worry that healthcare workers would give you poor care if you went to the CTC, because you are on MAT? |  |  |  |  |
| ADUMAT53 | If you were living with HIV, how often do you think you would worry that you would have to wait longer than other clients if you went to the CTC, because you are on MAT? |  |  |  |  |
| ADUMAT54 | If you were living with HIV, how often do you think you would worry that you would be judged if you went to the CTC, because you are on MAT? |  |  |  |  |
| ADUMAT55 | If you were living with HIV, how often do you think you would worry that you would be shamed if you went to the CTC, because you are on MAT? |  |  |  |  |
| ADUMAT56 | If you were living with HIV, how often do you think you would worry that you would be blamed if you went to the CTC, because you are on MAT? |  |  |  |  |
| ADUMAT57 | If you were living with HIV, how often do you think you would worry that you would be ignored if you went to the CTC, because you are on MAT? |  |  |  |  |
| ADUMAT58 | If you were living with HIV, how often do you think you would worry that healthcare workers would think that you have gone to steal from them if you went to the CTC, because you are on MAT? |  |  |  |  |
| ADUMAT59 | If you were living with HIV, how often do you think you would worry that health care workers would disclose to others that you are on MAT if you went to the CTC? |  |  |  |  |
| ADUMAT60 | If you were living with HIV, how often do you think you would worry that healthcare workers would verbally harass you (e.g., yell, scold, or otherwise verbally abuse) if you went to the CTC, because you are on MAT? |  |  |  |  |
| ADUMAT61 | If you were living with HIV, how often do you think you would worry if you do not dress properly, you would not be given care if you went to the CTC, because you are on MAT? |  |  |  |  |

**SECTION FOUR: PERCEIVED DRUG USE STIGMA**

I have asked you about your negative experiences with other people, as well as worries you have about how other people might think about or treat you, because you are on MAT. Now I want to ask you about what you think other people (the general population) think or assume about people who use drugs. The following section includes a series of statements that represent assumptions or views other people (people in the general population/ community) might have about people who use drugs. To answer these questions, please consider the thoughts, ideas, and impressions the general population has about people who use drugs. Please rate the extent to which you agree or disagree **that other people (people in the general population)** believe these statements, not the extent to which you personally believe these statements. Please tell me if you strongly disagree, disagree, neither disagree or agree, agree, or strongly agree that other people believe the following statements.

|  | **Item** | **Strongly disagree (0)** | **Disagree (1)** | **Neither disagree or agree (2)** | **Agree (3)** | **Strongly agree (4)** |
| --- | --- | --- | --- | --- | --- | --- |
| PDU1 | Most people believe that a person who uses drugs is dirty |  |  |  |  |  |
| PDU2 | Most people who use drugs are rejected when others find out that they use drugs |  |  |  |  |  |
| PDU3 | People who use drugs lose their jobs when their employers find out they use drugs |  |  |  |  |  |
| PDU4 | Most people think that a person who uses drugs is disgusting |  |  |  |  |  |
| PDU5 | Most people judge people who use drugs |  |  |  |  |  |
| PDU6 | Most people do not respect people who use drugs |  |  |  |  |  |
| PDU7 | Most people think people who use drugs have only themselves to blame for their drug use |  |  |  |  |  |
| PDU8 | Most people verbally harass (e.g., yell, scold) people who use drugs |  |  |  |  |  |
| PDU9 | Most people would willingly accept someone who has been treated for drug use as a close friend (not a sexual partner) |  |  |  |  |  |
| PDU10 | Most people believe that someone who has been treated for drug use is just as trustworthy as the average citizen |  |  |  |  |  |
| PDU11 | Most people would accept someone who has been treated for drug use as a teacher of children in a primary school |  |  |  |  |  |
| PDU12 | Most people would hire someone who has been treated for drug use to take care of their children |  |  |  |  |  |
| PDU13 | Most people think less of a person who has been in treatment for drug use |  |  |  |  |  |
| PDU14 | Most employers will hire someone who has been treated for drug use if he or she is qualified for the job |  |  |  |  |  |
| PDU15 | Most employers will pass over the application of someone who has been treated for drug use in favor of another applicant |  |  |  |  |  |
| PDU16 | Most people would be willing to marry someone who has been treated for drug use |  |  |  |  |  |

**INTERNALIZED DRUG USE STIGMA**

**The next set of questions are about feelings and thoughts you may have experienced because you are on MAT. Please rate the extent to which you agree or disagree with each of the following statements. Please select the answer that best reﬂects your thoughts and feelings today.** Please tell me if you strongly disagree, disagree, neither disagree or agree, agree, or strongly agree with the following statements.

| **Sn** | **Item** | **Strongly disagree (0)** | **Disagree (1)** | **Neither disagree or agree (2)** | **Agree (3)** | **Strongly agree (4)** |
| --- | --- | --- | --- | --- | --- | --- |
| IDUM1a | It is difficult to tell people that I was using drugs |  |  |  |  |  |
| IDUM1b | It is difficult to tell people that I am on MAT |  |  |  |  |  |
| IDUM6a | I now hide to people that I was using drugs |  |  |  |  |  |
| IDUM6b | I now hide to people that I am on MAT |  |  |  |  |  |
| 1DUM2 | Because I was using drugs, I now feel dirty |  |  |  |  |  |
| 1DUM3 | Because I was using drugs, I now I feel guilty |  |  |  |  |  |
| 1DUM4 | Because I was using drugs, I am now ashamed |  |  |  |  |  |
| 1DUM5 | I sometimes feel worthless because I was using drugs |  |  |  |  |  |

**ANTICIPATED HIV STIGMA**

In the following section I will ask you a series of statements about worries or concerns you might have about what other people think or may do, because you are living with HIV. **Please tell me whether the following experiences have never happened, happened once, a few times, or often.**

| **Sn** | **Statement** | **Never (0)** | **Once (1)** | **A few times (2)** | **Often (3)** |
| --- | --- | --- | --- | --- | --- |
| AH21 | If you were living with HIV, how often would you be worried that family members would avoid you because you are living with HIV? |  |  |  |  |
| AH22 | If you were living with HIV, how often would you be worried that family members would think that you cannot be trusted because you are living with HIV? |  |  |  |  |
| AH23 | If you were living with HIV, how often would you be worried that family members would look down on you because you are living with HIV? |  |  |  |  |
| AH24 | If you were living with HIV, how often would you be worried that family members will treat you differently because you are living with HIV? |  |  |  |  |
| AH25 | If you were living with HIV, how often would you be worried that your peers/other people who use or used drugs will avoid you because you are living with HIV? |  |  |  |  |
| AH26 | If you were living with HIV, how often would you be worried that your peers/other people who use or used drugs will gossip about you because you are living with HIV? |  |  |  |  |
| AH27 | If you were living with HIV, how often would you be worried that your peers/other people who use/used drugs will reject you because you are living with HIV? |  |  |  |  |
| AH28 | If you were living with HIV, how often would you be worried that people who knew you had HIV will tell others? |  |  |  |  |
| AH29 | If you were living with HIV, how often would you be worried that people would judge you when they learned you have HIV? |  |  |  |  |
| I am now going to ask you some questions about your experiences if you were living with HIV and you went to a hospital for general healthcare needs **that ARE NOT** related to **drug use or HIV treatment** like malaria, flu, or an accident. Please tell me whether the following experiences have never happened, happened once, a few times, or often. | | | | | |
| AH30 | If you were living with HIV, how often do you think you would worry that healthcare workers would not listen to your concerns when you went to a health facility for general health care because you are living with HIV? |  |  |  |  |
| AH31 | If you were living with HIV, how often do you think you would worry that healthcare workers would give you poor care when you went to a health facility for general health care because you are living with HIV? |  |  |  |  |
| AH32 | If you were living with HIV, how often do you think you would worry that you would be made to wait longer than other clients when you went to a health facility for general health care, because you are living with HIV? |  |  |  |  |
| AH33 | If you were living with HIV, how often do you think you would worry that healthcare workers would avoid physical contact with you when you went to a health facility for general health care because you are living with HIV? |  |  |  |  |
| AH34 | If you were living with HIV, how often do you think you would worry you would be disrespected when you went to a health facility for general health care because you are living with HIV? |  |  |  |  |
| AH35 | If you were living with HIV, how often do you think you would worry that you would be judged when you went to a health facility for general health care because you are living with HIV? |  |  |  |  |
| AH36 | If you were living with HIV, how often do you think you would worry that you would be shamed when you went to a health facility for general health care because you are living with HIV? |  |  |  |  |
| AH37 | If you were living with HIV, how often do you think you would worry that you would be blamed when you went to a health facility for general health care because you are living with HIV? |  |  |  |  |
| AH38 | If you were living with HIV, how often do you think you would worry that you would be ignored when you went to a health facility for general health care because you are living with HIV? |  |  |  |  |
| AH39 | If you were living with HIV, how often do you think you would worry that health care workers would disclose to others that you are living with HIV when you went to a health facility for general health care? |  |  |  |  |
| AH40 | If you were living with HIV, how often do you think you would worry that you will be verbally harassed (e.g., yelled ag, scolded, or otherwise verbally abused) when you went to the health facility for general health care because you are living with HIV |  |  |  |  |

**PERCEIVED HIV STIGMA**

The following section includes a series of statements that represent assumptions or views other people (people in the community) might have about persons who are living with HIV. To answer these questions, please consider the thoughts, ideas, and impressions the general population has about people who are living with HIV. Please rate the extent to which you agree or disagree that **other people believe these statements**, not the extent to which you personally believe these statements. Please tell me if you strongly disagree, disagree, neither disagree or agree, agree, or strongly agree that other people believe the following statements.

|  |  | **Strongly disagree (0)** | **Disagree (1)** | **Neither disagree or agree (2)** | **Agree (3)** | **Strongly agree (4)** |
| --- | --- | --- | --- | --- | --- | --- |
| PH1 | Most people believe that a person who has HIV is dirty |  |  |  |  |  |
| PH2 | Most people with HIV are rejected when others find out that they are living with HIV |  |  |  |  |  |
| PH3 | People with HIV lose their jobs when their employers find out they are living with HIV |  |  |  |  |  |
| PH4 | Most people think that a person with HIV is disgusting |  |  |  |  |  |
| PH5 | Most people judge people who are HIV positive |  |  |  |  |  |
| PH6 | Most people do not respect people who are HIV positive |  |  |  |  |  |
| PH7 | Most people think people living with HIV have only themselves to blame for being HIV positive |  |  |  |  |  |
| PH8 | Most people think people who are HIV positive are promiscuous |  |  |  |  |  |
| PH9 | Most people verbally harass (e.g., yell, scold) someone who is living with HIV |  |  |  |  |  |
| PH10 | Most people would willingly accept someone who is living with HIV as a close friend (not a sexual partner) |  |  |  |  |  |
| PH11 | Most people would accept someone who is living with HIV as a teacher of children in a primary school |  |  |  |  |  |
| PH12 | Most people would hire someone who is living with HIV to take care of their children |  |  |  |  |  |
| PH13 | Most people think less of a person who is living with HIV |  |  |  |  |  |
| PH14 | Most employers will hire someone who is living with HIV if he or she is qualified for the job |  |  |  |  |  |
| PH15 | Most employers will pass over the application of someone who is living with HIV in favor of another applicant |  |  |  |  |  |
| PH16 | Most people would be willing to marry someone who is living with HIV |  |  |  |  |  |

## 
