# Supplementary material for: Translation and cultural adaptation of drug use stigma and HIV stigma measures among people who use drugs in Tanzania
Source: PLoS One. 2023 Oct 19;18(10):e0292642. doi: 10.1371/journal.pone.0292642 (PMC10586607; doi:10.1371/journal.pone.0292642)
Supplement: Attachment — Submitted filename: Response to Reviewers.docx [file pone.0292642.s006.docx]

# **Translation and cultural adaptation of drug use stigma and HIV stigma measures among people who use drugs in Tanzania**

**Responses to Academic Editor’s comments**

| Sn | **Academic Editor’s comment** | **Response** | **Page number** |
| --- | --- | --- | --- |
| 1 | Please ensure that your manuscript meets PLOS ONE's style requirements, including those for file naming. | Thank you very much. We have ensured that our manuscript meets PLOS ONE’s style requirements including headings and supporting information. |  |
| 2 | Thank you for stating the following in the Acknowledgments Section of your manuscript:  "..This research was supported by the Fogarty International Center of the National Institute of Health (NIH), Award Number D43TW010946. The research content is solely the authors' responsibility, and the NIH had no role in study design, data collection, and analysis, decision to publish or preparation of the manuscript."  We note that you have provided funding information that is not currently declared in your Funding Statement. However, funding information should not appear in the Acknowledgments section or other areas of your manuscript. We will only publish funding information present in the Funding Statement section of the online submission form.  Please remove any funding-related text from the manuscript and let us know how you would like to update your Funding Statement. Currently, your Funding Statement reads as follows:  "The study was supported by the Fogarty International Center of the National Institutes of Health (NIH) under Award Number D43TW010946. The research is solely the responsibility of the authors, and the NIH had no role in study design, data collection and analysis, decision to publish, or preparation of the manuscript. No any, among authors have received salary from NIH."  Please include your amended statements within your cover letter; we will change the online submission form on your behalf. | We have removed the funding statement from the acknowledgments section.  We have added information in the Acknowledgements section about research assistants and participants.  We have included our Funding statement within the cover letter. | Please see this on page 30, Line 564 – 566. |
| 3 | Please include captions for your Supporting Information files at the end of your manuscript, and update any in-text citations to match accordingly. Please see our Supporting Information guidelines for more information: [http://journals.plos.org/plosone/s/supporting-information](http://journals.plos.org/plosone/s/supporting-information). | Thank you for the comment. We have added captions of Supporting Information files at the end of the manuscript and updated the in-text citations to match accordingly. | Please see this on page 9, Line 173, 176; on page 10, Line 206 – 208; and on page 33, Line 691 – 697. |
| 4 | Please review your reference list to ensure that it is complete and correct. If you have cited papers that have been retracted, please include the rationale for doing so in the manuscript text, or remove these references and replace them with relevant current references. Any changes to the reference list should be mentioned in the rebuttal letter that accompanies your revised manuscript. If you need to cite a retracted article, indicate the article’s retracted status in the References list and also include a citation and full reference for the retraction notice. | Thank you. The references have been reviewed and revised where there were errors. | Please see the revisions on page 30, Line 569 – 570, 575, 578 – 581; page 31, Line 589 – 594; and on page 32, Line 633 – 635, 645 – 650, 661 – 662. |
| 5 | I thank the authors for this very important and well written study. I agree with the reviewers' general evaluation that the manuscript requieres only minor revision before it is fit for publication. However, I recommend to pay close attention to the following issues, which may have also been pointed out by the reviewers: 1) Please state more clearly that this study does not yet include a psychometric evaluation of the scale (see also comment from reviewer 2), which is currently only mentioned at the end of the manuscript. | Thank you.  We are researching the stigma measures’ psychometric properties, and we will report the results separately. We have added this information to the manuscript. | Please see this in the Abstract on page 3, Lines 56 -58, in the Introduction on page 6, Lines 111 – 113, and in the main text on page 13, Line 276 – 278; and on page 30, Lines 555 – 556. |
| 6 | I really appreciate that the authors fully disclose all documents used in their study. However, concerning the strict data sharing policy of PLOS, I suggest to also share information on the translation of each item (original item -> translated item -> back translation) as well as the (english translated) transcripts from the interviews, since the cited responses and, thus, the qualitative evaluation strongly depend on that data. | Thank you. The information on the translation process is in the supplement (S1 File). We cannot share the participant transcripts because we did not ask for participants’ consent to share narrative data with an external third party. Upon a reasonable request, we can ask for permission from the IRBs for an exemption (contact Dr. Mlunde) |  |
| 7 | Please proofread the whole manuscript after revision to improve language quality as also suggested by reviewer 1. | Thank you. We have done this. |  |

**Responses to reviewers’ comments**

| Sn | **Reviewer’s comment** | **Response** | **Page number** |
| --- | --- | --- | --- |
|  | **Reviewer #1:** |  |  |
| 1 | The study presents the results of the original work. The steps of translation and adaptation of the scales have been explained in detail, making it easy for the reader to understand the process. | Thank you very much for your comment. |  |
| 2 | Important note: The study reports on the process of translating and adapting the scales and does not report the psychometric properties of the scales. When reporting the psychometric properties, it is recommended that each scale is discuss in a separate article as this will enable clear communication of the results. | Thank you. We are researching the stigma measures’ psychometric properties, and we will report the results separately. We have added this information to the manuscript. | Please see this in the Abstract on page 3, Lines 56 -58, in the Introduction on page 6, Lines 111 – 113, and in the main text on page 13, Line 276 – 278; and on page 30, Lines 555 – 556. |
| 3 | Method: The study follows Wild's 10 steps. It is recommended that the explanation of the steps and the details of the study (how the steps are used in the study) are reported together instead of discussing them separately as it leads to unnecessary confusions for the reader.  For example, Line 220-237 Sample size and sampling strategy of participants in the cognitive debriefing should be added with the explanation of step 8- Cognitive debriefing (line 196-199). | The steps have been reported together. We have combined all the details related to Step 8.  We have added this to Step 8 | Please see this on page 8, Lines 149 – 162.  Please see this on pages 11 – 11, Line 216 – 271. |
| 4 | Study design: Line 136- Authors have reported formative assessment, it is recommended that the mentioned formative assessment be discussed briefly and cited. | We have added the below text in the manuscript:  “The formative assessment was qualitative in design where it included in-depth interviews of 18 PWUD and 14 HIV care and treatment clinics (CTC) staff. The aim of the formative assessment was to understand the types of stigma and their manifestations that PWUD face. “ | Please see this on page 7, Line 142 – 145. |
| 5 | Six scales have been reported to be translated, it is recommended to add their brief overview | The brief overview is available on the table originally submitted. The information included was on measures’ types of stigma, number of items, and country of origin added. | Please see Table 1 on page 8. |
| 6 | Proofreading is recommended to clear a few language and typing errors | We have done proofreading to correct these errors. |  |
| 7 | Abbreviations mentioned should be written in full form the first time they are mentioned in the manuscript. | We have corrected this. |  |
|  |  |  |  |
|  | **Reviewer #2:** |  |  |
| 1 | There are a few typos through out, I would proofread this paper. | We have done proofreading to correct typos and improve clarity. |  |
| 2 | I commend the authors on the major task of 50 cognitive interviews. This far surpasses standard procedure and literature recommendations. It is a shame that the authors do not conduct psychometric analysis on a new sample, which would significantly enhance the paper. | Thank you very much.  We are researching the stigma measures’ psychometric properties, and we will report the results separately. We have added this information to the manuscript. | Please see this in the Abstract on page 3, Lines 56 -58, in the Introduction on page 6, Lines 111 – 113, and in the main text on page 13, Line 276 – 278; and on page 30, Lines 555 – 556. |
